# Research on Centroid Localization Method of Underground Space Ground Electrode Current Field Based on RSSI

**DOI:** 10.3390/s25092889

**Published:** 2025-05-03

**Authors:** Sirui Chu, Hui Zhao, Zhong Su, Xiangxian Yao, Xibing Gu, Yanke Wang, Zhongao Ling

**Affiliations:** 1 Beijing Key Laboratory of High Dynamic Navigation Technology, Beijing Information Science and Technology University, Beijing100192, China; 2023020380@bistu.edu.cn (S.C.); zhhui@bistu.edu.cn (H.Z.); 2023020388@bistu.edu.cn (X.Y.); 2023020390@bistu.edu.cn (X.G.); 2023020466@bistu.edu.cn (Y.W.); 2023020453@bistu.edu.cn (Z.L.); 2 Key Laboratory of Modern Measurement and Control Technology, Ministry of Education, Beijing Information Science and Technology University, Beijing 100192, China

**Keywords:** personnel localization, RSSI, centroid localization algorithm, received signal strength, ground electrode current field

## Abstract

Aiming to solve the problems of communication interruption caused by the collapse of underground space, this study constructs a strong penetration information transmission system and proposes a centroid localization method based on the received signal strength indication (RSSI) in an underground space ground electrode current field. This is applicable to localization in underground space such as subways, mines, tunnels, etc., as well as under the environment of collapse. First, the propagation characteristics of the ground current field signal in underground space are analyzed, and the attenuation model of the ground current field signal is constructed by combining the RSSI ranging method. On this basis, an improved weighted centroid localization algorithm is introduced to improve the localization accuracy and reliability by optimizing the algorithm parameters to cope with the fluctuations and instabilities generated in the signal propagation process. The experimental results show that the proposed localization method achieves an average positioning error of 7.47 m in an underground environment of 10,000 square meters, which is 32.32% less compared with the weighted centroid localization algorithm, and 62.74% less compared with the traditional centroid localization algorithm. This method presents a positioning technology that operates independently in underground spaces, overcoming the limitation of traditional wireless positioning systems, which rely on external transmission links. Its application will provide crucial technical support for life-saving operations in underground environments, acting as the ‘last line of defense’ in rescue missions. By completing the emergency response chain, it will enhance disaster rescue capabilities, offering substantial practical value and promising prospects.

## 1. Introduction

The core of autonomous localization of underground spaces (such as underground tunnels, mines, etc.) lies in the acquisition of two-dimensional plane coordinates. With the extensive development and utilization of underground space, underground places such as subways, mines, tunnels, etc., have gradually become important infrastructures of modern society. At the same time, the complexity and closure of underground space also bring many challenges in safety monitoring and personnel localization [1]. However, its special environmental characteristics, such as the complex geological structures, non-uniformity of signal propagation and multipath effects, make traditional wireless localization technology face many challenges in the underground environment, and there is an urgent need to develop localization methods that can operate effectively in the complex underground environment [2].

In recent years, with the deepening development and utilization of underground space, many scholars have carried out extensive research in the field of underground space positioning technology. Existing localization techniques such as ultra wide band (UWB), time difference of arrival (TDOA) and angle-of-arrival (AOA) perform well in surface environments, but are constrained by the complexity of the environment and the uncertainty of the signal propagation in underground space. These challenges limit the application of traditional wireless localization technologies in underground environments. Facing the problems of the multipath effect, complex geological structure and non-uniform attenuation of signal propagation in underground space, it is difficult to effectively guarantee the positioning accuracy and stability.

Among many localization methods, the localization method based on RSSI has gradually become one of the hotspots in the research of underground space localization, due to its advantages of easy implementation and low cost. Nowadays, most researchers are devoted to improving the practicality and reliability of RSSI positioning technology in underground space by constructing a more accurate signal attenuation model and optimizing algorithm parameters; applying RSSI technology to locate people in underground mines, tunnels and other environments, combined with wireless sensor networks (WSNs) to improve the positioning accuracy. Some researchers also introduce filtering algorithms, multipath effect correction models and other methods to mitigate the impact of signal interference on positioning accuracy. Chen et al. [3] propose a signal strength optimization algorithm based on Gaussian model, which further improves the accuracy and reliability of positioning by establishing an accurate RSSI signal strength distribution model and correcting abnormal RSSI values. Shahid et al. [4] present an experimental characterization of the proposed radio link model for an underground mine sensor network. The proposed radio link model was developed and evaluated for 433 MHz DASH7 in underground mines, considering the practical electromagnetic properties of mine walls and the propagation medium, which helps in calculating accurate signal characteristics. Radio wave propagation is a critical factor that needs to be considered when designing a wireless sensor network for complex mine structures. The radio link design has been optimized for complex mine structures by utilizing these parameters in a model, leading to improved performance and reliability. Mahmut et al. [5] investigate RSSI based localization techniques and apply them to indoor environments. A unique hybrid method based on fingerprint identification is proposed and developed to address the drawbacks of existing techniques. Vikram et al. [6] consider the RSSI-based self-localization problem for resource-constrained mobile nodes and propose a bias-compensated pseudo-linear solution (PLS) using a weighted least squares (WLS) approach. The weights are estimated using RSSI-induced distance estimation and the statistical properties of the perturbations in the anchor position observation. Ming et al. [7]. According to the engineering arrangement and operating environment characteristics of metal underground mines, field experiments of RSSI distance measurement were conducted in underground mines. Based on the experimental results, the RSSI-distance relationship model was established, and laboratory experiments of the BP neural network localization model were conducted. By using the combined localization model, the localization accuracy was improved and the interference of abnormal data was reduced. However, RSSI methods still face large technical challenges when dealing with underground complex environments, especially in scenarios where the signal propagation process is significantly affected by the multipath effect, and the localization accuracy is low, which restricts the widespread promotion of their practical applications.

For further improvement of RSSI positioning accuracy, the centroid localization method is proposed. RSSI centroid localization calculates the centroid of the target location by measuring the signal strength and using the geometric relationship of the nodes. However, in complex underground environments, RSSI values are greatly affected by signal attenuation and multipath effects, which leads to a decrease in the accuracy of centroid localization [8]. For this reason, Tahir et al. [9] present a new quadrant based weighted centroid algorithm solution, which incorporates alternating path loss factors based on the mining environment and uses RSSI indicators for distance calculation and improvement. Four beacon nodes were also used instead of the traditional three beacon nodes, and weights were applied to reflect the impact of each node on the centroid position. The weight factor applied is the reciprocal of the estimated distance. Hai et al. [10] propose an improved weighted centroid positioning algorithm based on RSSI to address the practical characteristics of the underground working environment in coal mines and the low positioning accuracy of existing algorithms. Modify the environmental parameters of RSSI ranging using the least squares method to eliminate the impact of various interferences on the measurement data. Directly calculate the index factor and the modified RSSI value to determine the coordinates of the unknown node.

Although these improved methods enhance the accuracy of RSSI centroid localization to a certain extent, the fully enclosed or nearly enclosed environment in the underground space leads to poor wireless signal transmission, or the transmission link even can be completely ‘cut off’. To cope with the problem, this paper proposes a strong penetration information transmission system under which the validation of all localization algorithms is carried out. This study proposes an RSSI-based ground electrode current field centroid localization method in underground space, which combines the characteristics of the ground current field signal with the RSSI ranging centroid localization algorithm to cope with fluctuations and instability during signal propagation and improve the localization accuracy and reliability. In this study, the concept of RSSI was applied to the propagation of current field signals in underground spaces. Unlike traditional RSSI measurement of received signal strength in radio wave propagation, in this study RSSI refers to the strength of extremely low frequency current signal propagated through underground media. This study provides an independent and highly reliable technical solution for personnel localization and emergency rescue in underground space, which has important application prospects and practical value. Meanwhile, this research provides theoretical foundation and technical support for further exploration and optimization of positioning technology in underground space.

The rest of this paper is organized as follows: Section 2 introduces the design and calibration of the strong penetration information transmission system based on ground electrode current field. Section 3 describes the weak signal detection process and signal preprocessing techniques. Section 4 presents the RSSI-based distance estimation model and the parameter fitting method. Section 5 proposes the improved weighted centroid localization algorithm. Section 6 provides the experimental results and comparative analysis with other algorithms. Finally, Section 7 concludes the paper and discusses potential future work.

## 2. Strong Penetrating Information Transmission System

When a collapse occurs in an underground environment, it usually results in the formation of a fully enclosed collapse body that traps people and ‘cuts off’ the external signal transmission link. In order to locate the trapped person when the communication link is ‘cut off’, this study proposes a strong penetration information transmission system,

### 2.1. System Introduction

**Figure 1:**
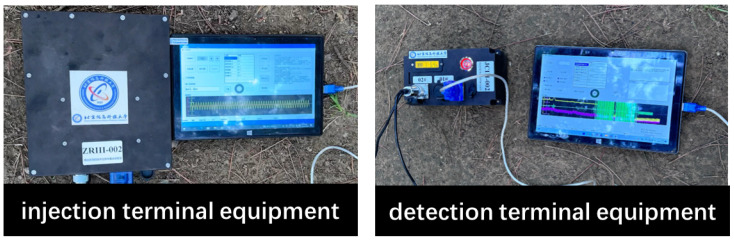
Signal injection terminal equipment, detection terminal equipment.

The system uses an extremely low frequency [11] current field signal, transmitted through two sets of electrodes arranged in the underground space: one set at the injection end and the other at the detection end. The equipment at the injection end, shown in Figure 1, is connected to the electrodes via wires. The device at the injection end injects the modulated ground current signal into the underground space, forming a controllable ground current field signal through the ground electrode. Its internal components mainly include a signal source module, a power supply system and an injection control unit. Among them, the signal source module is used to output preset waveform signals, which are injected into the underground medium by the ground electrode to achieve strong penetration of information transmission. The detection device is used to receive transmission signals at other locations in the underground space, and it integrates a signal acquisition module, detection filtering module, synchronous positioning module and data processing and recording module internally. After filtering, the received signal is synchronously processed and amplitude and other information are extracted for subsequent positioning models. This equipment injects signals, which flow into the ground through the electrodes, with the ground serving as the medium for signal transmission. The detection end then measures the ground current field signals, enabling long-distance signal transmission [12].

**Figure 2:**
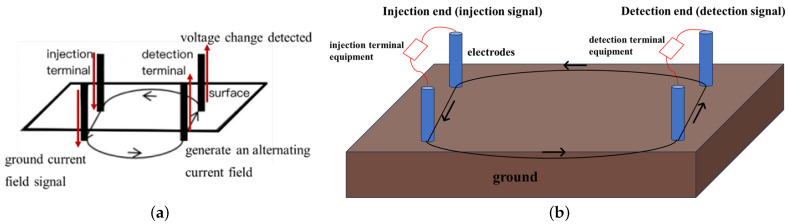
(**a**) Schematic diagram of horizontal ground current field signal transmission. (**b**) Schematic diagram of the system operation (injection and detection of signals).

**Figure 3:**
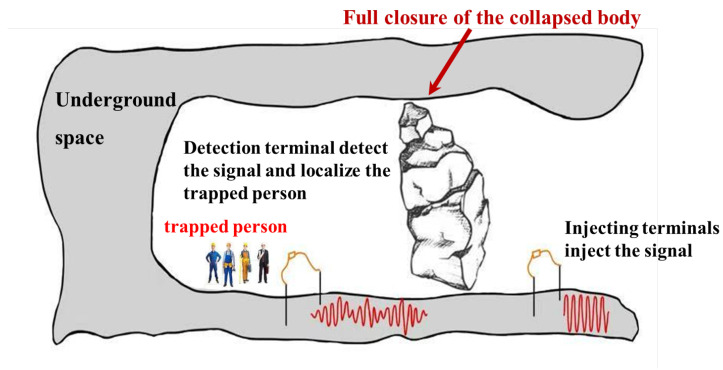
Schematic diagram of the actual application scenario.

The signal transmission principle is shown in Figure 2a,b. The schematic diagram of the practical application in underground space is shown in Figure 3. In this application scenario, the trapped person injects signals into the underground space by burying the electrodes at the detection end into the ground, while an external person injects the signals into the underground space through the equipment at the injection end. The trapped person receives the current field signals transmitted underground at the detection end, and processes the detected signals as well as estimates, and determines the position based on the localization method proposed in this study. The design and layout of the system take into account the complexity and non-uniformity of the underground environment, so that the signal can penetrate through a variety of geologic media, such as rock layers, collapsed bodies, sandy soil and reinforced concrete structures.

**Figure 4:**
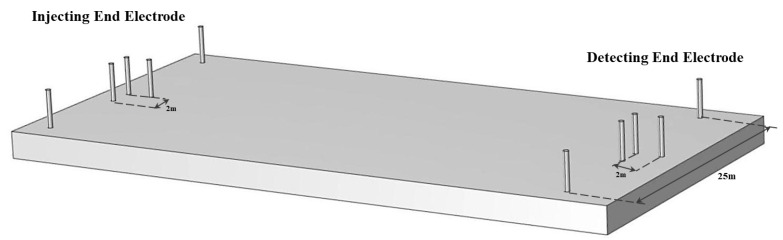
System Electrode Arrangement.

The core hardware module of the system includes the signal injection end, signal detection end, electrode system and signal transmission line. The signal injection end and detection end devices are shown in Figure 1. The experimental data involved in this paper are derived from the actual measurement results of the constructed strong penetration information transmission system. The ground current field signal during the operation of the system was collected through several experiments, according to which the subsequent detection of effective signals, the calculation of signal strength and the localization of relevant data were carried out. The signal injection end adopts the double electrode injection design, which injects an extremely low frequency current field signal into the underground space through two electrodes, thus forming a stable ground electrode current field. Compared with the traditional single-electrode design, the dual-electrode layout effectively reduces the signal attenuation problems caused by poor contact between electrodes and unreasonable distance, and enhances the signal transmission strength and uniformity. In practice, the system realizes signal injection and detection through the electrodes at the injection end and detection end buried in the underground space. The electrode arrangement shown in Figure 4 clearly shows the relative positions of the signal injection and detection ends. The signal detection end adopts an “L” type three-electrode structure in order to capture the voltage changes of the underground signal sensitively. By increasing the detection dimension between the electrodes, the structure significantly improves the anti-interference of signal detection, and is able to detect current changes more effectively in complex geological environments with serious signal attenuation, thus ensuring the accuracy and stability of signal reception. The electrodes at the injection and detection ends were buried at a depth of 0.5 m, with the electrode spacing at the injection end set at 25 m and the electrode spacing at the detection end set at 2 m to improve the accuracy of the voltage signals. Each electrode has a length of 1.2 m and a radius of 0.015 m, ensuring a sufficient contact area.

**Figure 5:**
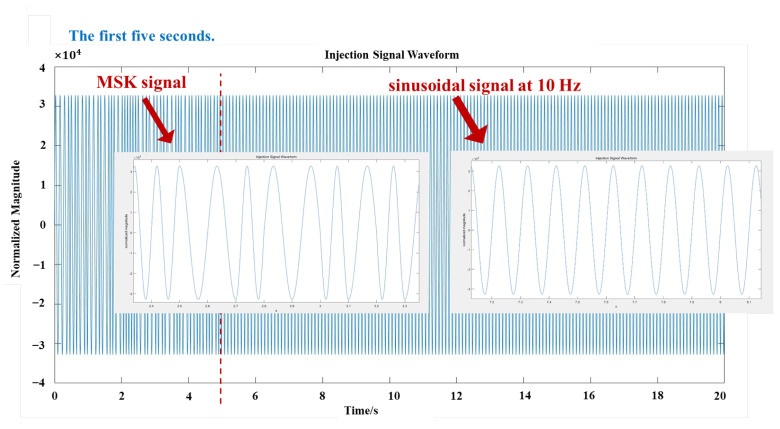
The waveform of the injected signal.

In terms of enhancing the penetration capability of the system, this system has been optimized from various aspects such as signal selection, adaptive design and anti-interference measures. First of all, extremely low frequency current field signals have lower attenuation in the underground medium, and can be less affected by rock and soil and other media. The injection side of the system injects a ground current field signal with a total duration of 15 s, the first five seconds being an MSK signal for matching and the last ten seconds being a sinusoidal signal at a frequency of 10 Hz, at an injection voltage of 100 V to ensure sufficient propagation strength in the complex underground environment. The waveform of the injected signal is shown in Figure 5. Secondly, in order to adapt to the design of the ground electrode current field in various geological environments, the system is designed for the resistance difference of geological media such as rock, sandy soil, collapsed body, reinforced concrete, etc. By adjusting the electrode layout and the value of the injection voltage, the system makes the ground electrode current field adaptable to the demand for signal transmission under different geological conditions, and ensures the ability to penetrate in different media. To further enhance the anti-interference capability of the system, the design introduces a band-pass filter to filter out the noise in the non-target frequency band, and time-aligns the received signal with the known injected signal through the signal synchronization module to calculate the correlation of the signals to ensure that the detected signal is the target signal. In addition, the detection end adopts a dual-channel signal capture design, through the “L” type three electrodes to obtain two signal channels, the signal-to-noise ratio needs to reach 10 dB or more before retaining the data. The multi-channel screening strategy significantly improves the reliability of the data and reduces the impact of invalid noise data on the system accuracy.

In the process of signal transmission, the signal injection end injects an extremely low frequency current field signal into the underground space through double electrodes, thus forming a ground electrode current field in the underground medium, and then captures and detects the voltage change of the signals through the three-electrode structure at the detection end to realize the strong penetration of information transmission [13,14,15]. The ground current field signal can penetrate different geological media and effectively realize the long-distance transmission of the signal. Experimental data show that the system can still maintain effective signal transmission in the complex underground environment, which lays a technical foundation for the realization of underground space localization system.

### 2.2. System Calibration

**Figure 6:**
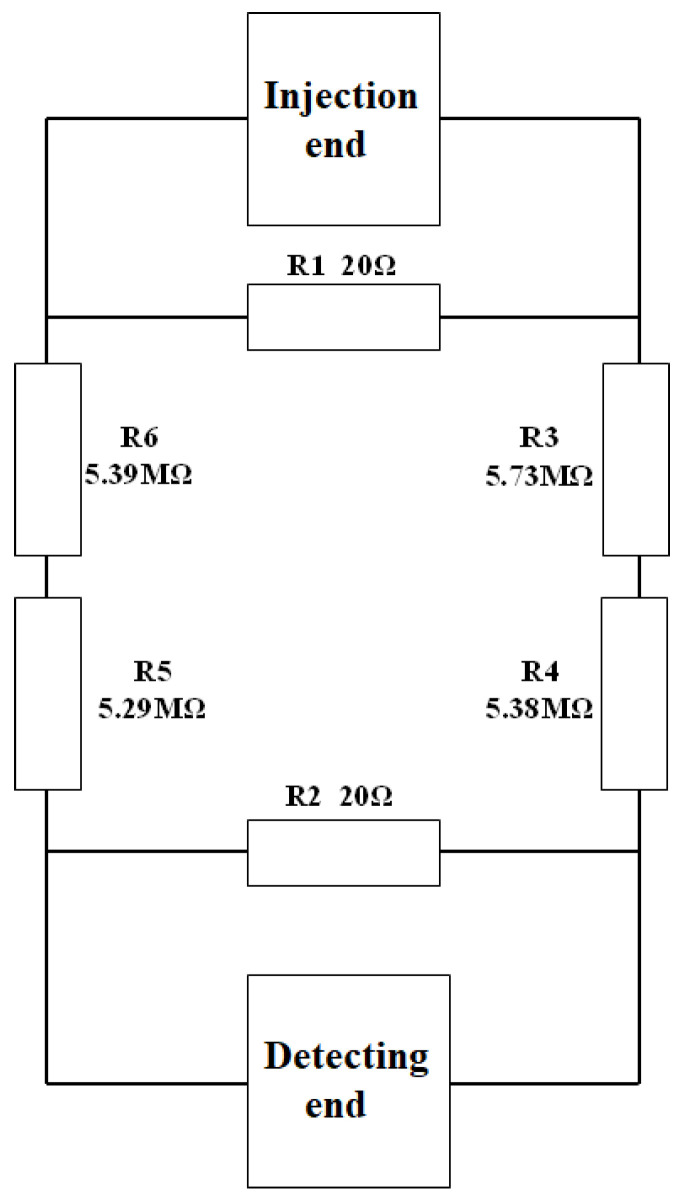
Equivalent Circuit.

In order to ensure the accuracy of the experimental system, it is necessary to calibrate the system before the experiment. The calibration adopts the calibration method of the equivalent resistance network. We simulate the electrical characteristics of the earth using an equivalent resistance network composed of several resistance elements. The purpose is to simulate the transmission characteristics of the ground electrode current field in underground space. The equivalent circuit diagram is shown in the Figure 6. The resistance values are obtained through experiments and theoretical calculations to ensure accurate simulation of ground resistance characteristics.

On this basis, according to Ohm’s law, given different injection voltages (5 V, 10 V, 15 V, *…*, 50 V), the voltage value at the output terminal can be calculated as the true value through an equivalent resistance network. We measure using different versions of prototype machines (prototype numbers: ZRIII-001, ZRIII-002, ZRIII-003, ZRIII-004) under the same circuit connection. The injected signal at each voltage level will undergo multiple tests and the average value will be taken to reduce measurement fluctuations. We obtain the voltage values at each test point using instruments. The core step of calibration is to calculate the deviation between the measured value and the theoretical value. The specific method is to compare the measured values at each voltage level with the corresponding theoretical values to obtain the measurement error. Due to the fact that the error is roughly a multiple relationship, it can be corrected by a correction coefficient (multiple). The formula is as follows:

(1) $$V_{measured}=k*V_{theoretical}.$$

Among them, *k* is the correction coefficient (used to represent the proportional relationship between the measured value and the true value), which remains a single constant during the calibration process, representing the ratio between the measured value and the theoretical value. By calculating the *k* values at different voltages and averaging them, a unified correction factor can be obtained for correcting other measurement results. By using this correction coefficient, all subsequent measurement data can be corrected, thereby improving the measurement accuracy of the positioning system. This calibration process ensures that the difference between the measured values and the true values of our system is minimized, thereby improving the positioning accuracy and reliability of the system in underground environments.

#### 2.2.1. Calibration of Injection End

**Figure 7:**
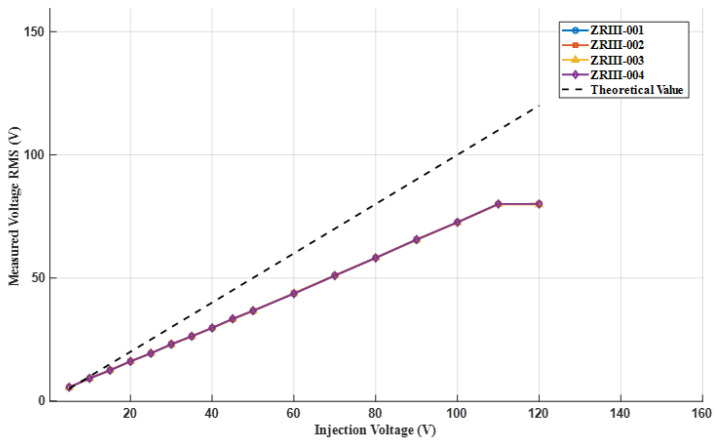
The average effective value of injection voltage for different injection ends.

This experiment calibrated four current injection ends (ZRIII-001 to ZRIII-004). We measure the effective output voltage of each end using an oscilloscope. In the experiment, each set of injected voltage signals was measured twice and considered as one set of data. A total of 85 sets of data measurements were conducted, of which 11 sets had voltage fluctuations exceeding 1%. After retesting, it was confirmed that these abnormal fluctuation data do not affect the overall accuracy of the measurement, and the voltage effective value fluctuations of all terminals in both measurements were controlled within 1%. Due to the fact that the effective voltage measurement fluctuation of the oscilloscope is less than 1% when the injection voltage signal is set to be the same, the mean of each set of data is selected as the final measurement result. The measurement result curve is shown in Figure 7.

#### 2.2.2. Calibration of Detection End

**Figure 8:**
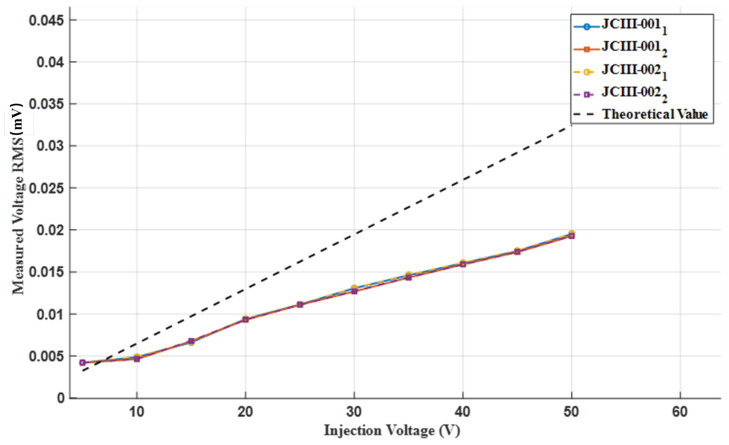
Measured/theoretical values of detection voltage under different injection voltages.

This experiment calibrated two current detection ends (prototype number: JCIII-001, JCIII-002). When using a prototype for testing, three signals are injected for each voltage level, including channel 1 (JCIII-001/002$d_{1}$) and channel 2 (JCIII-001/002$d_{2}$), for a total of 30 × 4 signals. The maximum standard deviation of the 30 sets of signals is 0.000229, indicating that the voltage fluctuations during the three tests at each voltage level are not significant. Therefore, the average value is taken. The measurement result curve is shown in Figure 8, which represents the theoretical effective value.

The results show that the effective values of the output voltage of the four stage current injection ends are highly consistent among different devices, and the measurement errors are all controlled within 1%, indicating that the devices exhibit good performance in output stability and consistency. At each set injection voltage, the corresponding calibration values are obtained by calculating the average output voltage of each device. These values serve as the standard output voltage of the device, providing a reliable reference for subsequent response performance verification. Further calibration and testing results indicate that the output voltage of different signal channels of each device is stable under different injection voltages, further verifying the reliability of the device under different working conditions. In summary, the tested system is capable of stable response under various working conditions and meets the calibration requirements in practical applications.

## 3. Detection of Weak Signals at the Detection End

In complex underground environments, weak signal detection is a key challenge to achieve effective information transmission. The underground space is full of various noise sources, such as power line interference, natural electromagnetic fluctuations and interference from underground equipment and other man-made sources. These noises significantly affect the quality of signal reception, making the effective signal easily masked by background noise.

Weak signal detection is based on the known correlation between the injected and detected signals, and the target signal is extracted by time alignment and filtering. Two sets of electrodes are set up in the test as the injection and detection ends of the ground current field signal. The detection end first receives a mixed signal, which contains the target signal and environmental noise. Before the injection signal is sent, the detection end receives mainly ambient noise; while after the injection signal starts, the detection end receives a superposition of the target signal and noise. To extract the effective signal, the system preprocesses the received mixed signal, including signal denoising and synchronization.

The signal denoising process employs a band-pass filter to filter out the noise components in the non-target frequency bands and retain the frequency bands of the injected signal. The design of the filter needs to incorporate the spectral characteristics of the noise in the subsurface environment and select the appropriate frequency band to enhance the denoising effect. To ensure the accuracy of the signal processing process, the system has designed a signal synchronization module to align the received signal with the known injected signal in time. This module adopts the sliding window correlation analysis method, using the injected signal as a reference template, and sliding point by point in the received signal to calculate its correlation coefficient. By finding the maximum value of the correlation coefficient, it is possible to accurately locate the starting position of the target injection signal in the received signal, thereby achieving synchronous alignment. This method effectively suppresses noise interference, improves the accuracy of signal extraction and provides a reliable data basis for subsequent field strength extraction and positioning operations. The synchronization module is particularly suitable for identifying and extracting weak signals in complex underground environments, and is a key link in ensuring the overall accuracy of the system.

**Figure 9:**
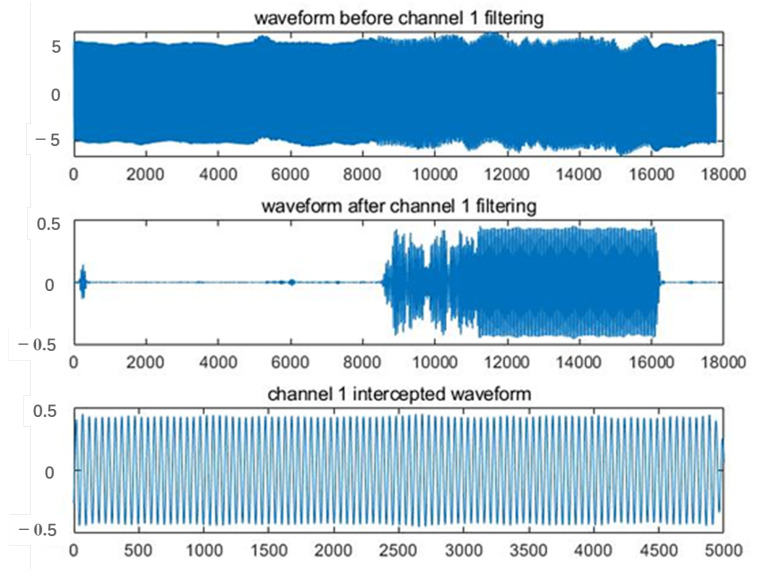
Effective waveform after filtering and intercepting.

After signal pre-processing and synchronization, the system performs effective signal interception of the detected signal and uses it for subsequent analysis. This part of the extracted signal can be used for precise positioning or other data analysis after further processing. The detected valid signal is shown in Figure 9.

## 4. RSSI Ranging Model

The RSSI ranging and positioning method is widely used in practical ranging and positioning due to its advantages of low cost, low power consumption, simple usage, relatively easy deployment and no need for time synchronization. In most cases, RSSI is used for measuring the transmission of ground radio waves, but in this study, RSSI was applied to the transmission of underground current field signals. Unlike the definition of RSSI for electromagnetic waves, we estimate the target distance by measuring the strength of the underground current field signal. This signal propagates through geological media such as rock layers, sandy soil, etc., and the propagation process is influenced by various factors such as geological media and geological interference.

### 4.1. Modeling

The RSSI ranging and positioning method estimates the transmission loss between the sending point and the receiving point by the strength of the received data signal, and then converts the transmission loss between the two points into the distance between the two points according to the theoretical model. Currently, the theoretical model commonly used for wireless signal transmission is the shadowing model. The model formula is as follows [16,17,18,19,20]:

(2) $$PL\left(d\right)=PL\left(d_{0}\right)-10n\mathrm{lg}\left(\frac{d}{d_{0}}\right)+X,$$

where $d_{0}$ is the reference distance; *d* is the distance between the electrode at the injection end and the electrode at the detection end; *n* is the signal transmission constant and *n* varies in different environments; $PL(d_{0})$ is the received signal strength at the distance $d_{0}$ between the injection end and the detection end; *X* is a Gaussian random variable with a mean value of 0 and a mean squared deviation of $\sigma_{dB}$ (dB); PL(d) is the received signal strength at the detection end.

For experiments in practical environments, the actual model was selected as follows [21,22]:

(3) $$RSSI=A-10n\mathrm{lg}\left(\frac{d}{d_{0}}\right),$$

where the RF parameter *A* is the absolute value of the average energy of the detection end at a reference distance from the injection end, that is, the signal strength received at the detection end at a distance of $d_{0}$ from the injection end, which varies in different environments; *d* is the distance between the injection end and the detection end; and RSSI is the received signal strength at the detection end. From this, the measured distance *d* can be obtained as [23]

(4) $$d=d_{0}*10^{\frac{A-RSSI}{10n}}.$$

### 4.2. Acquisition of Fitting Parameters

When performing RSSI ranging, signal transmission is susceptible to environmental noise and ground resistance. To ensure data accuracy and positioning reliability, the received signal must be rigorously screened. This process includes screening for signal-to-noise ratio (SNR) and ground resistance to exclude invalid data that may affect ranging accuracy.

In the complex environment of underground space, the signal may be interfered by various noises during transmission, resulting in a significant degradation of signal quality. Based on empirical observations from previous experiments, the signal-to-noise ratio (SNR) threshold was empirically set to 10 dB as a minimal value to ensure data reliability. Any data with an SNR lower than 10 dB was considered invalid and excluded from further processing. The detection end adopts the ‘L’ type three-electrode arrangement, and the two sides are respectively used as two channels to collect signals through dual-channel detection. Therefore, the final signal-to-noise screening needs to meet the signal-to-noise ratio of each channel, and the combined signal-to-noise ratio of the two channels are greater than 10 dB.

The specific operation of the detection consists of calculating the signal amplitude and noise amplitude for each data set, and the signal-to-noise ratio for a single channel as well as the combined signal-to-noise ratio for a two-channel set are calculated by the following equation:

(5) $$SNR_{signal\;channel}=20*\log_{10}\frac{P_{signal}}{P_{noise}},$$

(6) $$SNR_{combined\;signal-to-noise\;ratio}=\frac{\sqrt{P_{signal\;channel1}2+P_{signal\;channel2}2}}{\sqrt{P_{noise\;channel1}2+P_{noise\;channel2}2}},$$

where $P_{signal}$ and $P_{noise}$ denote the signal amplitude and noise amplitude, respectively. The calculation of the combined signal-to-noise ratio takes into account the signal and noise of both channels to improve the accuracy of the data.

In the underground environment, the ground resistance varies greatly, and if the resistance value is too high, it will lead to serious signal attenuation, thus affecting the accuracy of the positioning results. Based on the experimental experience, the screening range of ground resistance is set to be 0 Ω ∼ 500 Ω. Data exceeding this range will be regarded as invalid data and be rejected.

After the screening of signal-to-noise ratio and ground resistance, the quality of the screened data is significantly improved, which reduces the influence of environmental noise and stratigraphic complexity on the localization results, thus laying a foundation for the subsequent calculation of RSSI fitting parameters.

When performing RSSI ranging, the selection of fitting parameters *A* and *n* is crucial for the accuracy of the ranging results. To optimize these parameters, a linear regression algorithm is typically used to fit the experimental data [21]. Assuming that $RSSI_{i}$ and $d_{i}$ are experimental measurements, where $RSSI_{i}\;\left(i=1,2,3,...,k\right)$ represents the signal strength at distance $d_{i}\;\left(i=1,2,3,...,k\right)$. *k* is the number of signal strength sampling points, the parameters *A* and *n* can be calculated by linear regression analysis using the following equation:

(7) $$n=\frac{\sum_{i=1}^{k}\left(\rho_{i}-\bar{\rho }\right)RSSI_{i}}{\sum_{i=1}^{k}{\left(\rho_{i}-\bar{\rho }\right)}^{2}},$$

(8) $$A=\bar{RSSI}-\bar{\rho },$$

in the formula:

(9) $$\bar{\rho }=\frac{1}{k}\sum_{i=1}^{k}\rho_{i},$$

(10) $$\bar{RSSI}=\frac{1}{k}\sum_{i=1}^{k}RSSI_{i}.$$

**Figure 10:**
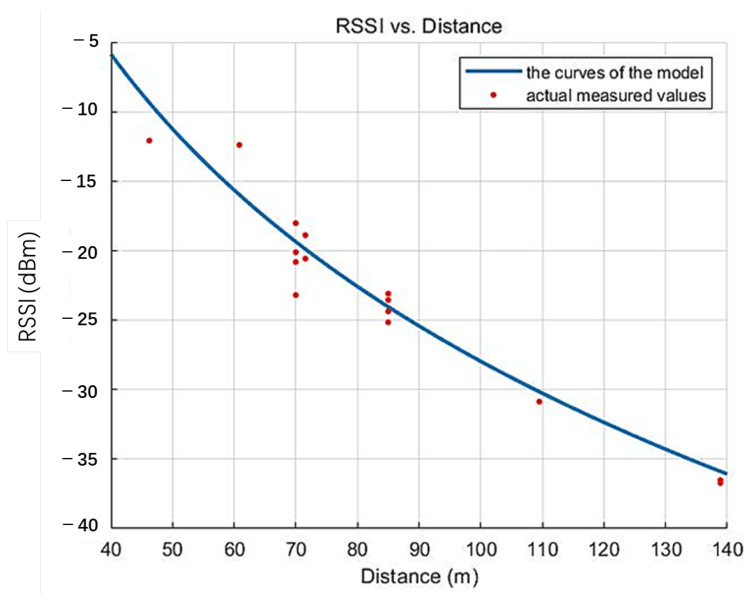
RSSI versus distance.

Taking the underground alleyway environment as an example, experimental tests were carried out in the distance range of 70 m to 140 m, and 16 sets of data were measured, which were substituted into the above equation to obtain *A* = −19.4 and *n* = 5.6. Figure 10 shows the model curve of the distance loss coefficient of the RSSI measured data versus, which can be well fitted to the current environment according to the linear regression analysis [24].

In addition, the fitting process needs to consider the influence of environmental factors, such as the humidity, temperature and geological features of the experiment environment, which will cause different degrees of interference and attenuation to the signal transmission. Therefore, when optimizing the parameters, several measurements should be carried out to obtain comprehensive data, so as to improve the reliability and high applicability of the fitting results.

## 5. Localization Algorithm

Trilateral localization is one of the commonly used node localization methods in wireless sensor networks. Its basic idea is to determine the localization of the unknown node through geometric computation by using the distance information between the beacon node and the unknown node with known position. The ideal trilateral localization algorithm is able to precisely locate the coordinates of the target node under the assumption that the beacon node position is known and the measured distance is accurate.

**Figure 11:**
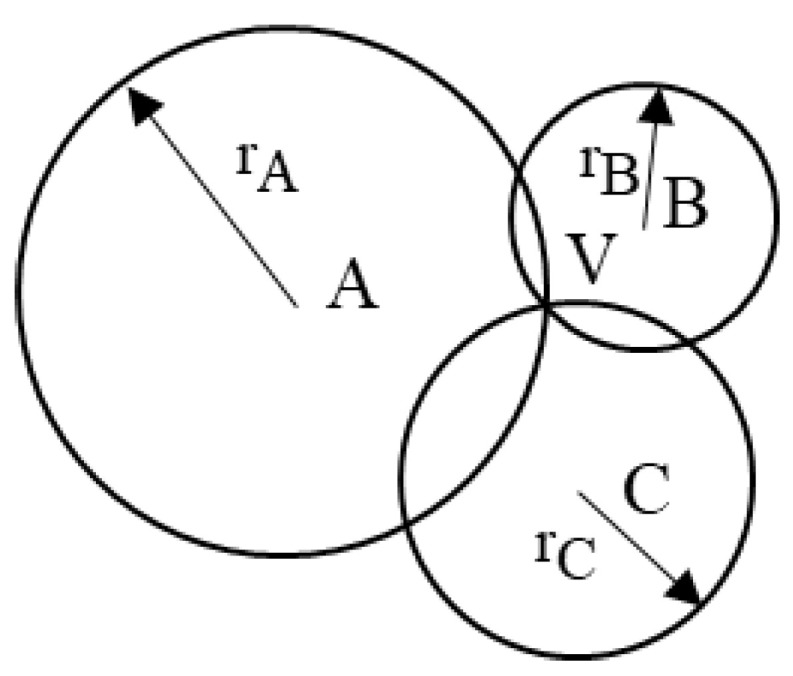
Schematic diagram of the localization node in the ideal case.

In the two-dimensional plane, assume that three beacon nodes, *A*, *B* and *C*, with known locations are used to determine the location of an unknown node *V*. As shown in Figure 11, the coordinates of the three known beacon nodes are $A(X_{A},Y_{A})$, $B(X_{B},Y_{B})$ and $C(X_{C},Y_{C})$, and the coordinate of the node at the unknown location is $V(X_{V},Y_{V})$. At the same time, the distances of the node *V* from the three beacon nodes *A*, *B* and *C* are known to be $d_{A}$, $d_{B}$ and $d_{C}$, respectively.

According to the Euclidean distance formula, the position of node *V* satisfies the following system of equations:

(11) $$\left\{\begin{array}{c} \sqrt{{\left(X_{V}-X_{A}\right)}^{2}+{\left(Y_{V}-Y_{A}\right)}^{2}}=d_{A}\\ \sqrt{{\left(X_{V}-X_{B}\right)}^{2}+{\left(Y_{V}-Y_{B}\right)}^{2}}=d_{B}\\ \sqrt{{\left(X_{V}-X_{C}\right)}^{2}+{\left(Y_{V}-Y_{C}\right)}^{2}}=d_{C} \end{array}\right..$$

By solving the above system of equations, the coordinates of the unknown node *V* can be obtained $\left(X_{V},Y_{V}\right)$.

### 5.1. Trilateral Centroid Localization

In practical applications, signals are often affected by environmental factors during transmission, such as signal attenuation, multipath effects and noise interference, leading to errors in distance measurement. These errors make the node position calculated by the ideal trilateral localization algorithm no longer a precise point, but a region, increasing the uncertainty of localization. Therefore, trilateration centroid localization algorithm is commonly used for localization in practical applications.

The trilateration centroid localization algorithm is characterized by higher accuracy and simplicity of the algorithm. Based on the concept of the geometric center, the unknown node is assumed to be located at the centroid of the polygon formed by the beacon nodes, and the coordinate values of multiple beacon nodes with known coordinates are averaged to estimate the location of the unknown node.

**Figure 12:**
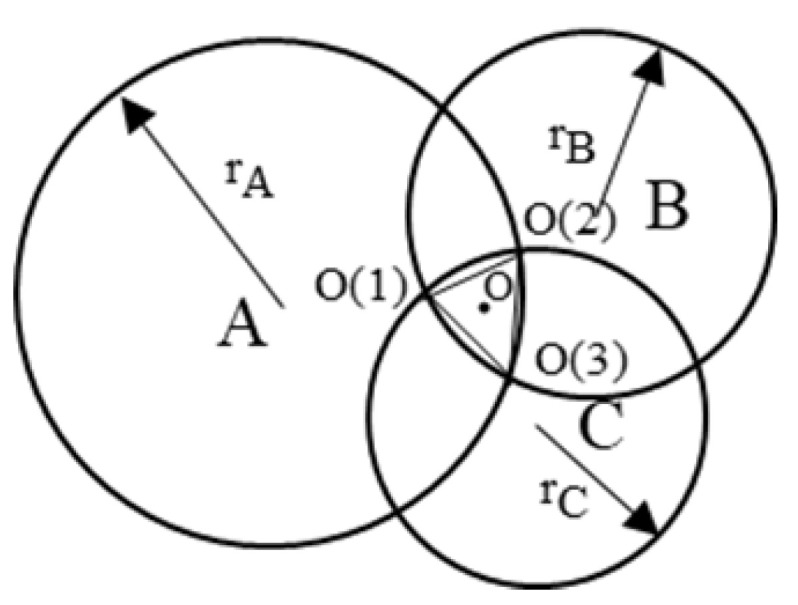
Schematic diagram of the trilateral centroid localization nodes.

There are three beacon nodes *A*, *B*, *C* and an unknown node *O* in the plane, as shown in Figure 12, with coordinates $\left(X_{A},Y_{A}\right)$, $\left(X_{B},Y_{B}\right)$, $C(X_{C},Y_{C})$, and the location of the target unknown node *O* can be estimated by averaging the coordinates of the three beacon nodes.

The triangular vertices that can be used for localization are O(1), O(2) and O(3). The coordinates of the unknown node *O* can be replaced by the centroid of ΔO(1)O(2)O(3). The coordinates of the unknown node *O* can be expressed as follows:

(12) $$\left(\frac{X_{O(1)}+X_{O(2)}+X_{O(3)}}{3},\frac{Y_{O(1)}+Y_{O(2)}+Y_{O(3)}}{3}\right).$$

### 5.2. Weighted Centroid Localization

In the traditional centroid localization algorithm, the influence of the reference points on the target point is simply averaged, i.e., the centroid position is directly averaged from the coordinates of all the reference points. However, in practice, due to the gradual attenuation of the signal strength with the increase in the distance, reference points farther away tend to contribute less to the localization results, while reference points closer to the target have a greater impact on the localization results. Therefore, the traditional centroid algorithm is easily affected by reference points far away from the target point, resulting in localization results that deviate from the actual target.

In the weighted centroid algorithm, each beacon node is given a different weight based on its distance from the unknown node, and the closer the node, the greater the weight, thus affecting the final localization result to a greater extent. The weights are usually proportional to the signal strength, measurement accuracy or other factors related to the localization accuracy.

**Figure 13:**
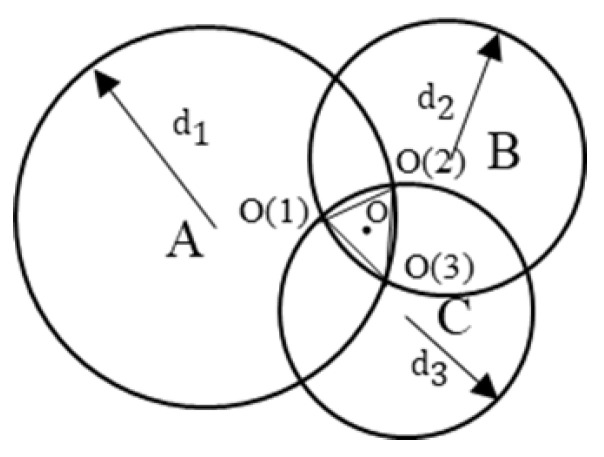
Schematic diagram of weighted centroid localization nodes.

As shown in Figure 13, for the three known locations $A(X_{A},Y_{A})$, $B(X_{B},Y_{B})$ and $C(X_{C},Y_{C})$, and and their corresponding weights $\omega_{1}$, $\omega_{2}$ and $\omega_{3}$, the coordinates of the target point *o* can be calculated by weighted average:

(13) $$\left(\frac{\omega_{1}X_{A}+\omega_{2}X_{B}+\omega_{3}X_{C}}{\omega_{1}+\omega_{2}+\omega_{3}},\frac{\omega_{1}Y_{A}+\omega_{2}Y_{B}+\omega_{3}Y_{C}}{\omega_{1}+\omega_{2}+\omega_{3}}\right).$$

Circle *A* and circle *B* intersect at point O(3), the radius of the two circles are $d_{1}$, $d_{2}$, point O(3) corresponding to the weights of $\frac{1}{d_{1}+d_{2}}$, point O(1) and point O(2), and so on, then the coordinates of the target point *O* expressed in terms of weights:

(14) $$\left(\frac{\frac{X_{A}}{d_{1}+d_{2}}+\frac{X_{B}}{d_{2}+d_{3}}+\frac{X_{C}}{d_{1}+d_{3}}}{\frac{1}{d_{1}+d_{2}}+\frac{1}{d_{2}+d_{3}}+\frac{1}{d_{1}+d_{3}}},\frac{\frac{Y_{A}}{d_{1}+d_{2}}+\frac{Y_{B}}{d_{2}+d_{3}}+\frac{Y_{C}}{d_{1}+d_{3}}}{\frac{1}{d_{1}+d_{2}}+\frac{1}{d_{2}+d_{3}}+\frac{1}{d_{1}+d_{3}}}\right).$$

The weighted centroid localization algorithm integrally considers the weights of each localization point, which significantly improves the localization accuracy compared with the ordinary centroid localization algorithm, but it is still limited by the measurement noise, the simplicity of the signal attenuation model and the complexity of the environmental factors in the practical application.

### 5.3. Centroid Localization in a Ground Electrode Current Field

The weights used in the weighted centroid algorithm often simply use the reciprocal of the distance, which still fails to fully reflect the effect of distance on signal attenuation in some scenarios. To further improve the localization accuracy, an improved weighting method is proposed on the basis of the weighted centroid algorithm. In this improved algorithm, the weight of the reference point is no longer just the sum of the reciprocal of the distance, but the sum of the reciprocal of the distance squared is used as the weight, and the power value is added to improve it, which makes the distribution of the distance weight more reasonable:

(15) $$\frac{1}{d_{1}+d_{2}}\to \frac{1}{d_{1}^{n}}+\frac{1}{d_{2}^{n}}.$$

**Figure 14:**
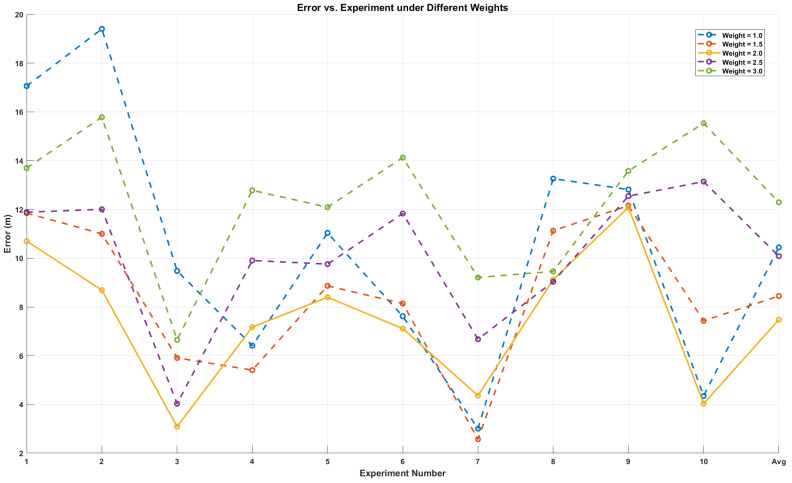
The error of different weights in the improved weighted centroid localization algorithm.

By adding power values to the weights as mentioned above, the situation where secondary data information has the main impact is eliminated. Assuming $d_{1}<d_{2}$, it indicates that the anchor nodes located at an unknown distance from node $d_{2}$ only play a secondary role in the weights. However, when power values are not added for correction, they actually play a primary role, masking the main role played by $d_{1}$ in the weights. By adding power values to the weights, the main role played by $d_{1}$ in the weights was restored. The n in the weight is the correction coefficient. The weight of information was reasonably arranged by adjusting the size of the weights. After multiple experimental verifications, the accuracy is higher when the weight value is set to 2. The positioning errors of different weights are shown in Figure 14. The weight value is as follows:

(16) $$\frac{1}{d_{1}+d_{2}}\to \frac{1}{d_{1}^{2}}+\frac{1}{d_{2}^{2}}.$$

Suppose that there exists an unknown node that captures the signals of three beacon nodes and the positions of beacon nodes *A*, *B* and *C* are $\left(X_{A},Y_{A}\right)$, $\left(X_{B},Y_{B}\right)$ and $\left(X_{C},Y_{C}\right)$, respectively, and the estimated distances between the unknown node and the beacon nodes are $d_{1}$, $d_{2}$ and $d_{3}$, respectively, and the coordinates of the improved weighted centroid *G* are expressed in terms of weights:

(17) $$\left(\frac{\frac{X_{A}}{\frac{1}{d_{1}^{2}}+\frac{1}{d_{2}^{2}}}+\frac{X_{B}}{\frac{1}{d_{2}^{2}}+\frac{1}{d_{3}^{2}}}+\frac{X_{C}}{\frac{1}{d_{1}^{2}}+\frac{1}{d_{3}^{2}}}}{2\cdot (\frac{1}{d_{1}^{2}}+\frac{1}{d_{2}^{2}}+\frac{1}{d_{3}^{2}})},\frac{\frac{Y_{A}}{\frac{1}{d_{1}^{2}}+\frac{1}{d_{2}^{2}}}+\frac{Y_{B}}{\frac{1}{d_{2}^{2}}+\frac{1}{d_{3}^{2}}}+\frac{Y_{C}}{\frac{1}{d_{1}^{2}}+\frac{1}{d_{3}^{2}}}}{2\cdot (\frac{1}{d_{1}^{2}}+\frac{1}{d_{2}^{2}}+\frac{1}{d_{3}^{2}})}\right).$$

## 6. Analysis of Experimental Results

**Figure 15:**
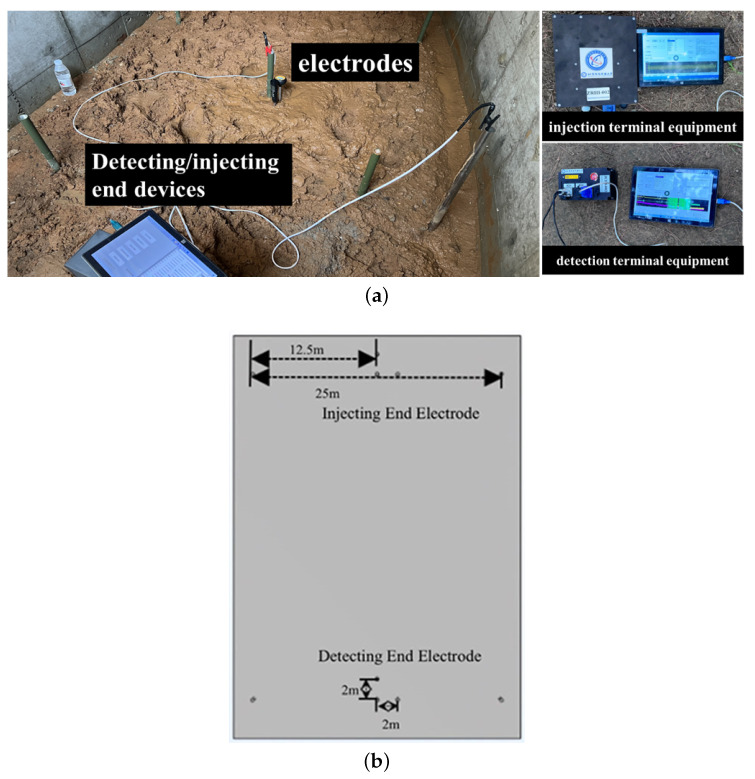
(**a**) Electrodes laid out at the experimental site and equipment at the injection and detection ends. (**b**) Electrode layouts at the injection and detection ends plotted on the simulation software.

In this study, a strong penetration information transmission system was constructed, which is represented in Figure 2 in Section 2. The system was designed to transmit a signal from a known end to an unknown end. It consists of a known injection end (signal transmitting device), an unknown detection end (signal detecting device), wires, and 10 heels of electrodes with a length of 1.2 m and a radius of 0.015 m. Three groups of known injectors and one group of unknown detectors are deployed. The system adopts two-electrode injection, ‘L’ type three-electrode detection layout for ground current field signal transmission. The injection end injects a extremely low frequency ground current field signal, an alternating ground electrode current field is formed between the two electrodes, and the detection end detects the voltage change to realize the signal transmission. The distance between the two electrodes at the injection end is 25 m, and the buried depth is 0.5 m. The distance between the two electrodes at the detection end is 2 m, and the buried depth is 0.5 m. The electrode layout of the experimental site, as well as the equipment and electrodes, are shown in Figure 15a,b. The injection end injects a 15 s total duration ground current field signal, the first five seconds is a MSK signal for matching, and the last ten seconds is a sinusoidal signal with a frequency of 10Hz, and the injection voltage is 100 V.

The experimental tests were conducted in a typical underground sheltered space, which is fully enclosed and incapable of receiving natural light or satellite signals, providing excellent electromagnetic shielding. The spatial structure consists of concrete walls, a steel-structured ceiling, and a reinforced ground surface. Certain sections are filled with gravel and moist soil to simulate the heterogeneous media commonly found in real underground environments. The testing area includes infrastructure such as metal pipelines and cable ducts, forming complex signal propagation paths. This environment is characterized by significant signal attenuation and reflection.

**Figure 16:**
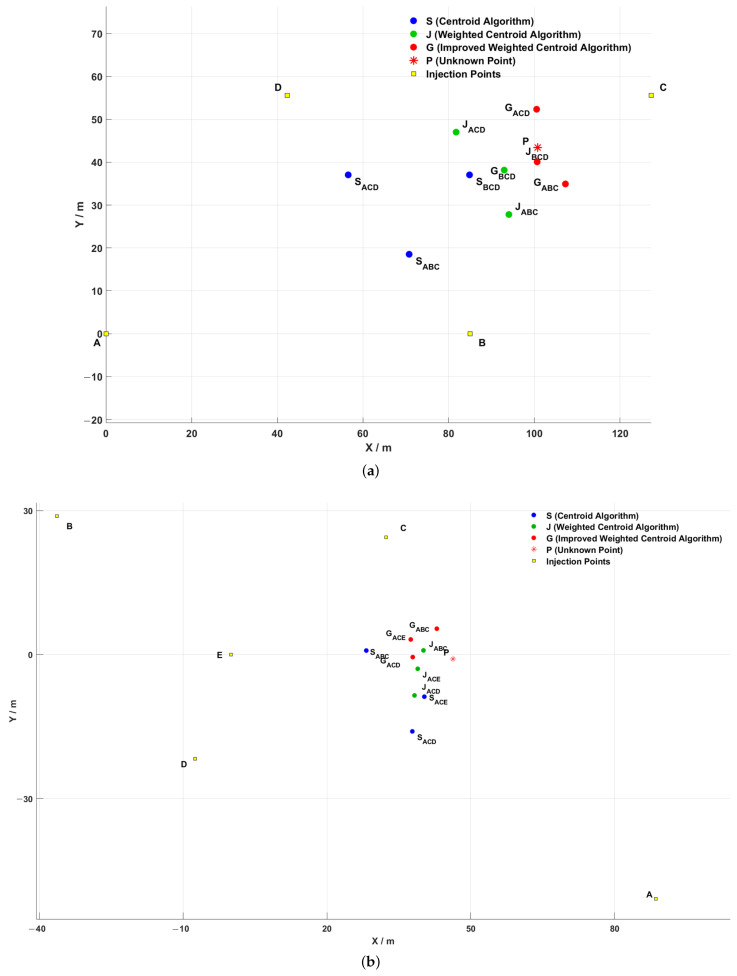

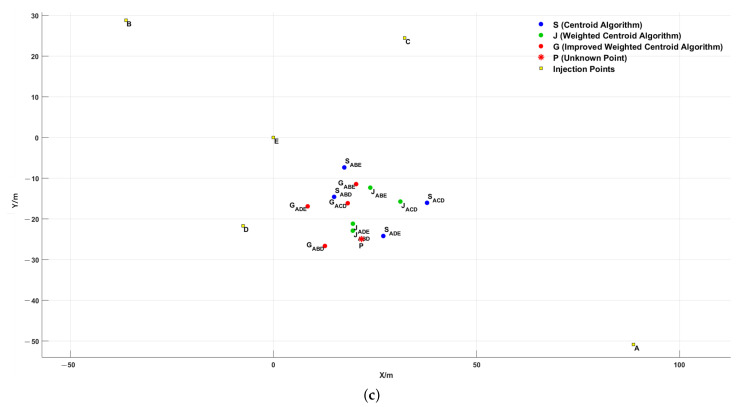
(**a**–**c**) Three different unknown detection ends *P* and the localization results of each algorithm.

A, B, C, D, E are the deployed injection terminals, these five injection terminals are freely combined, and each time three injection terminals inject signals, which are detected at the same unknown detection terminal. Three unknown detection points to be localized are randomly deployed in different areas, and the following localization experiments are carried out respectively in weather environments with different temperature and humidity. The localization results are shown in Figure 16, where for example, ACE means three injection points, injection point A, injection point C and injection point E, are used as three beacon nodes for centroid localization, and $G_{ACE}$ is the unknown localization node for improved weighted centroid localization. The other points are the same.

**Figure 17:**
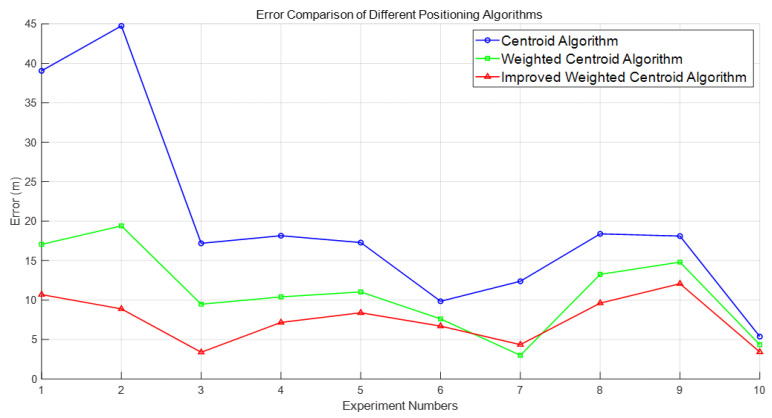
Localization errors of three localization algorithms.

Table 1 shows the ambient temperature and formation humidity for each experiment. In the actual experimental process, natural changes in temperature and humidity are inevitable in the testing environment, which may have a certain impact on the conductivity of underground media and equipment performance, thereby causing fluctuations in signal amplitude or noise level. However, by comparing and analyzing the positioning errors under different temperature conditions, it can be found that the proposed positioning method still exhibits good stability and consistency in multiple experiments, with overall small fluctuations in errors and no significant offset. This indicates that the system has a certain robustness to environmental changes, can maintain high positioning accuracy in actual complex working conditions, and has good engineering application potential. The localization errors of the obtained centroid positions of each algorithm, with respect to the unknown detected points and the comparison of the errors between the algorithms, are shown in Figure 17. In the figure, the blue circle is the centroid localization result, the green circle is the weighted centroid localization result, and the red circle is the improved weighted centroid localization result. Table 2 shows the error ranges of three positioning algorithms, including mean error, minimum error and maximum error.

It can be seen from Figure 13 that through the positioning experiment, within the positioning range of the experiment, the traditional centroid algorithm does not consider the distance difference between the reference point and the target point when dealing with the positioning task of the underground space, resulting in low positioning accuracy. Especially in complex environments, reference points far away from the target have a greater impact on the localization results, leading to larger localization deviations. In contrast, the weighted centroid algorithm significantly improves the localization accuracy by introducing distance weights. Reference points closer to the target have a more significant impact on the localization results, and the higher the weight, the higher the contribution to the final localization accuracy. However, although the weighted centroid algorithm has a reduced impact on reference points far away from the target, since the algorithm simply uses the inverse of the sum of distances for weight assignment, error fluctuation still occurs in some experimental scenarios, suggesting that there is still room for improvement of the algorithm in complex environments.

In order to further enhance the localization accuracy, the improved algorithm proposed in this paper makes the weight allocation more reasonable by using the sum of the inverse of the squares of the distances as the weights. The experimental results show that the error between the centroid position obtained by the improved weighted centroid algorithm and the unknown detection point is 7.47 m. The positioning distance errors of the improved weighted centroid algorithm are almost all smaller than those of the original weighted centroid algorithm, and the positioning accuracy is improved by 32.35%, and the errors are all smaller than those of he traditional centroid localization algorithm, and the positioning accuracy is improved by 62.74%, which is obviously better than the other two algorithms. The improved algorithm and the strong penetration information transmission system can better cope with the problems of communication link interruption caused by the collapse of underground space, especially in the fully enclosed environment to show greater reliability and independence.

## 7. Conclusions

In this study, an improved weighted centroid localization method based on RSSI is proposed for problems such as communication disruption caused by underground space collapse. Under the strong penetrating information transmission system, the ground current field signal transmission attenuation feature is used for localization. Through experimental verification, the improved algorithm demonstrates higher localization accuracy and robustness in complex environments. Compared with the traditional centroid and weighted centroid algorithms, the improved weighted centroid algorithm has a more significant localization effect in underground space, which effectively reduces the localization error caused by multipath effects and signal fluctuation. The improved RSSI-based ground electrode current field localization method in underground space has a wide range of application prospects and is suitable for personnel localization and emergency rescue in complex underground spaces such as subways, mines and tunnels.

It should be emphasized that the method presented in this article is a preliminary study aimed at exploring the application of ground electrode current field in underground space localization. Although research on the ground electrode current field has gradually begun, its application for underground localization is still in a relatively preliminary stage. This method draws inspiration from the RSSI positioning method. In the future, if the optimization of localization algorithms, improvement of electrode layout, adjustment of injected signal parameters, and optimization of localization feature selection can be combined, it is expected to further improve the localization accuracy. Relevant research will be the focus of subsequent work.

## Figures and Tables

**Table 1:** Temperature and humidity conditions of the ten experimental sites.

Experiment Times	1	2	3	4	5	6	7	8	9	10
Temperature (°)	32.8	31.7	29.4	24.5	27.7	26.4	24.4	20.1	15.6	13.3
Humidity (%)	54.7	33.6	33.1	64.6	55.8	53.1	74.4	75.3	79.1	77.8

**Table 2:** The error range of three positioning algorithms.

Localization Algorithm	Mean Error (m)	Minimum Error (m)	Maximum Error (m)
Centroid algorithm (m)	20.06	5.38	44.75
Weighted centroid algorithm (m)	11.04	3.00	19.40
Improved Weighted centroid algorithm (m)	7.47	3.39	12.08

## Data Availability

The data presented in this study are available in the article.

## Abbreviations

The following abbreviations are used in this manuscript:

RSSI	Received signal strength indicator
SNR	Signal-to-Noise Ratio
